# Specific Thiazolidinediones Inhibit Ovarian Cancer Cell Line Proliferation and Cause Cell Cycle Arrest in a PPARγ Independent Manner

**DOI:** 10.1371/journal.pone.0016179

**Published:** 2011-01-21

**Authors:** Linah Al-Alem, R. Chase Southard, Michael W. Kilgore, Thomas E. Curry

**Affiliations:** 1 Department of Molecular and Biomedical Pharmacology, University of Kentucky College of Medicine, Lexington, Kentucky, United States of America; 2 Department of Obstetrics and Gynecology, University of Kentucky College of Medicine, Lexington, Kentucky, United States of America; Florida International University, United States

## Abstract

**Background:**

Peroxisome Proliferator Activated Receptor gamma (PPARγ) agonists, such as the thiazolinediones (TZDs), have been studied for their potential use as cancer therapeutic agents. We investigated the effect of four TZDs—Rosiglitazone (Rosi), Ciglitazone (CGZ), Troglitazone (TGZ), and Pioglitazone (Pio)—on ovarian cancer cell proliferation, PPARγ expression and PPAR luciferase reporter activity. We explored whether TZDs act in a PPARγ dependent or independent manner by utilizing molecular approaches to inhibit or overexpress PPARγ activity.

**Principal Findings:**

Treatment with CGZ or TGZ for 24 hours decreased proliferation in three ovarian cancer cell lines, Ovcar3, CaOv3, and Skov3, whereas Rosi and Pio had no effect. This decrease in Ovcar3 cell proliferation was due to a higher fraction of cells in the G_0_/G_1_ stage of the cell cycle. CGZ and TGZ treatment increased apoptosis after 4 hours of treatment but not after 8 or 12 hours. Treatment with TGZ or CGZ increased PPARγ mRNA expression in Ovcar3 cells; however, protein levels were unchanged. Surprisingly, luciferase promoter assays revealed that none of the TZDs increased PPARγ activity. Overexpression of wild type PPARγ increased reporter activity. This was further augmented by TGZ, Rosi, and Pio indicating that these cells have the endogenous capacity to mediate PPARγ transactivation. To determine whether PPARγ mediates the TZD-induced decrease in proliferation, cells were treated with CGZ or TGZ in the absence or presence of a dominant negative (DN) or wild type overexpression PPARγ construct. Neither vector changed the TZD-mediated cell proliferation suggesting this effect of TZDs on ovarian cancer cells may be PPARγ independent.

**Conclusions:**

CGZ and TGZ cause a decrease in ovarian cancer cell proliferation that is PPARγ independent. This concept is supported by the finding that a DN or overexpression of the wild type PPARγ did not affect the changes in cell proliferation and cell cycle.

## Introduction

Ovarian cancer is the fifth leading cause of cancer death in women. Of the three main types of ovarian cancer (epithelial, germ cell, and sex cord stromal cancers), epithelial ovarian cancer accounts for about 90% of all cases, and is the first cause of death from gynecological malignancies [1], [2]. Despite intense research on ovarian cancer with new targets being constantly investigated, treatment targets remain sparse. One of the challenges in ovarian cancer research is the absence of an experimental animal model that recapitulates the human disease that can be experimentally manipulated [1], [3]. Thus, ovarian cancer cell lines have been employed to understand the fundamental processes involved in cancer cell growth, differentiation, and proliferation. The present study utilized three ovarian cancer cells, Ovcar3, CaOv3 and Skvo3, which are derived from human epithelial ovarian cancer [4], [5] to further explore therapeutic modalities in cancer cell growth and proliferation.

One of the therapeutic targets under investigation for ovarian cancer is nuclear receptors. Drugs that activate or inhibit nuclear receptors have been used to treat many diseases. Indeed, about 13% of the drugs currently on the market target nuclear receptors [6]. PPARγ is a highly conserved nuclear receptor [7] expressed throughout the body [8] and is over expressed in many cancers, including ovarian and breast cancer, making it a potentially important player in the development of cancer.

Endogenous PPARγ ligands are still unknown, but well characterized candidates include polyunsaturated fatty acids, Prostaglandin J_2_ (PGJ_2_) and arachidonic acid [9]. Synthetic PPARγ ligands include the thiazolidinediones (TZDs), which consist of Rosiglitazone (Avandia®), Troglitazone (Rezulin®), Pioglitazone (Glustin ®/Actos®), and Ciglitazone, all of which have been developed and/or used to treat type II diabetes [10], [11], [12]. The use of TZDs as a therapeutic approach in cancer has been investigated but results have been controversial [13], [14], [15]. In this study we utilized molecular, physiological and pharmacological approaches to investigate the effect of the four different TZDs on ovarian cancer cells and determine whether these effects are PPARγ dependent or independent.

## Results

### Ovcar3, CaOv3 and Skov3 ovarian cancer cell lines express PPARγ

**Figure 1:**
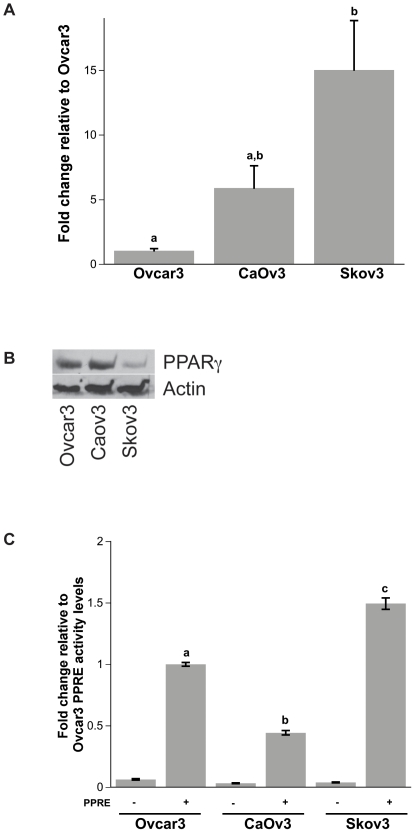
Expression of PPARγ in three ovarian cancer cell lines (Ovcar3, CaOv3, Skov3). (**A**) mRNA expression (**B**) Protein expression and (**C**) PPRE luciferase activity. Data was normalized to levels of PPARγ expression and activity in Ovcar3 cells. Results are the means ± SEM for at least 3 measurements from three individual experiments. Bars that do not share a letter designation are significantly different (*p*<0.05). Cells transfected with Luc and Renilla constructs lacking PPRE (PPRE −) were significantly lower than the same cell line transfected with PPRE-Luciferase-Renilla constructs (PPRE +) by Welch's t-test (*p*<0.05).

In order to determine whether ovarian cancer cells express PPARγ, real time PCR and western blot analysis was performed. There was differential PPARγ expression in the three different cell lines both at the mRNA and protein levels. While PPARγ mRNA expression was highest in Skov3 cells (Figure 1A), Skov3 cells had the lowest PPARγ protein levels (Figure 1B). In contrast, Ovcar3 had low levels of PPARγ mRNA expression but abundant expression of PPARγ protein (Figures 1A and 1B respectively). PPARγ activity in the three cell lines was examined using cells transfected with a 3XPPRE-Luc-*Renilla* construct and compared to cells transfected with luciferase and *Renilla* constructs lacking the PPRE. Ovcar 3 cells exhibited approximately 2 fold more endogenous PPRE activity compared to CaOv3 cells, while Skov3 cells showed 50% more PPRE activity compared to Ovcar3 cells (Figure 1C).

### Retinoic Acid Receptor (RXR) is not a limiting factor in PPARγ activity

**Figure 2:**
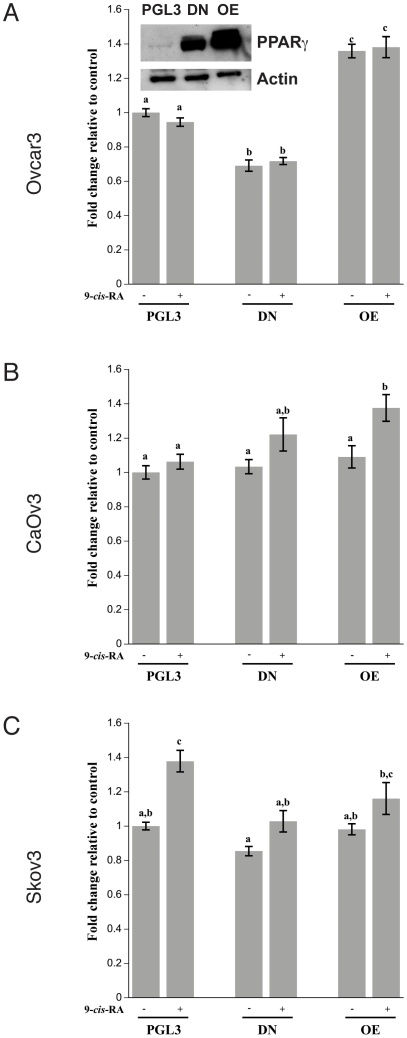
Effect of 9-*cis*-Retinoic Acid (9-*Cis*-RA) treatment on Ovcar3, CaOv3 and Skov3 PPRE luciferase activity. Cells were double transfected with a PPRE construct and one of the following: Empty vector (PGL3), DN form of PPARγ (DN) or wildtype form of PPARγ (OE). After the double transfections, cells were treated for 24 hours with vehicle control (DMSO) or 1 µM of 9-*Cis*-RA in the dark. PPRE luciferase activity is illustrated for (**A**) Ovcar3, (**B**) CaOv3, and (**C**) Skov3. Controls represent cells that were transfected with the empty vector and treated with vehicle control, shown as the first bar in each panel. Results for the PPRE luciferase assay are the means ± SEM for at least 9 measurements. Bars that do not share a letter designation are significantly different (*p*<0.05). Insert **Panel A**: Western blot of PPARγ protein in Ovcar3 cells double transfected with a PPRE construct and either empty vector, DN or OE PPARγ constructs and treated with vehicle control for 24 hours.

PPARγ binds to its heterodimeric partner RXR before binding to DNA [16]. RXR is known to be constitutively expressed in cells and has been shown to heterodimerize with other receptors besides PPARγ, such as the vitamin D receptor [17]. For RXR to be activated, its ligand 9-*cis*-retinoic acid (9-*Cis*-RA) needs to be present. In order to insure that activated RXR is not a limiting factor in our experiments, cells were treated with 9-*Cis*-RA. In these experiments, cells were transfected with different PPARγ constructs including Δ467 which is a dominant negative (DN) form of PPARγ [18], [19] as well as an over expression form of wild type PPARγ (OE) which is used to increase PPARγ expression. A DN form of PPARγ was used throughout these studies based upon initial experiments that revealed that DN transfection decreased the PPRE activity 40% below levels in cells transfected with ShRNA PPARγ (data not shown). In order to insure that PPARγ was overexpressed whether in the DN or OE form compared to control cells (i.e. cells transfected with PGL3), PPARγ protein expression was measured using western blot analysis. As expected there was a marked induction of both the DN and OE form of PPARγ (Figure 2A insert). After transfection with the control, DN or OE vector, cells were subsequently treated with vehicle control or 1 µM of 9-*Cis*-RA for 24 hours in the dark. We observed that the presence of 9-*Cis*-RA did not affect the activity of the PPARγ reporter assay in Ovcar 3 cells, indicating that activated RXR is not a limiting factor in this cell line (Figure 2A). The addition of 9-*Cis*-RA to CaOv3 cells did not change PPRE activity until PPARγ was overexpressed (Figure 2B) suggesting that activated RXR is not rate limiting until PPARγ is highly abundant in this cell line. Surprisingly, in Skov3 cells, 9-*Cis*-RA increased the PPARγ reporter activity assay in control cells (PGL3) but had no effect when PPARγ was over expressed compared to control (Figure 2C).

### CGZ and TGZ cause a decrease in cell proliferation

**Figure 3:**
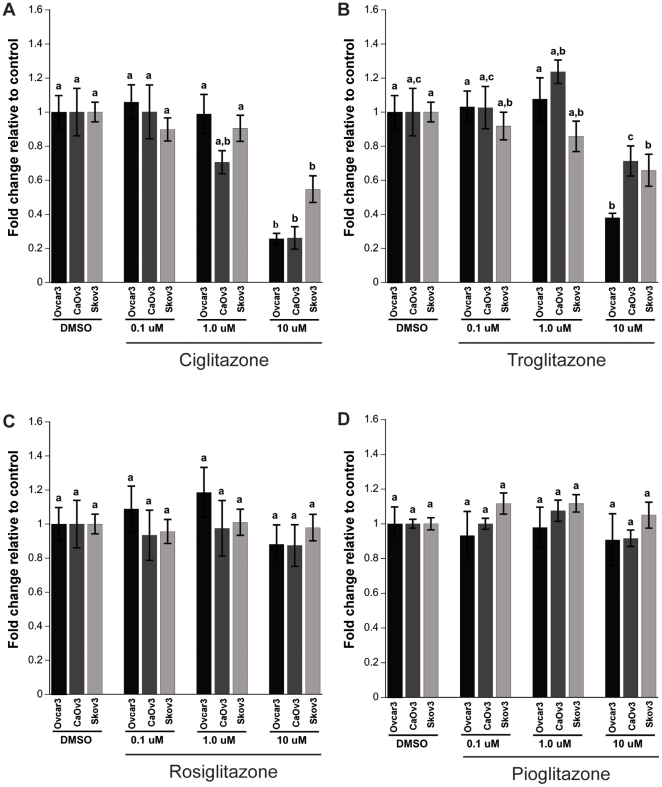
Effect of TZD treatment on Ovcar3, CaOv3 and Skov3 cell proliferation. Ovarian cancer cells were serum starved for 24 hours and treated for an additional 24 hours with vehicle control (DMSO) or 0.1, 1.0 or 10 µM of CGZ (**A**), TGZ (**B**), Rosi (**C**), or Pio (**D**). Cell proliferation was assessed with the MTS assay. Results are the means ± SEM for at least 3 measurements from three individual experiments. Statistical analysis was performed within each cell line. Bars that do not share a letter designation are significantly different within a treatment group (*p*<0.05). Black bars: Ovcar3, Grey: CaOv3, Light grey: Skov3.

To define the effects of PPARγ ligands on cell proliferation, MTS assays were performed. Ovcar3 cells were treated with concentrations of 0, 0.1, 1 or 10 µM of Rosi, CGZ, TGZ or Pio for 24 hours. The maximum TZD concentration used was 10 µM since PPARγ specific actions are reported with TZDs at concentrations ≤10 µM [20] and higher concentrations are known to induce cell death [21]. Administration of the four TZDs resulted in differences in cell proliferation. Treatment with CGZ decreased proliferation 80%, TGZ caused a 65% decrease, while Rosi and Pio had no effect on proliferation (Figure 3A–D Black bars). In an effort to understand whether the decrease in proliferation following TZD treatment is common across other ovarian cancer cells, TZD treatment of CaOv3 and Skov3 was explored. A similar decrease in proliferation was seen in these cell lines when treated with CGZ and TGZ at 10 µM. CaOv3 cells show an 80% and 30% decrease in cell proliferation when treated with CGZ and TGZ, respectively (Figure 3A–B). Skov3 cells show a 45% and 35% decrease when treated with 10 µM CGZ and TGZ, respectively (Figure 3A–B). Similar to Ovcar3 cells, CaOv3 and Skov3 cells treated with Rosi or Pio did not exhibit a change in proliferation (Figure 3C–D).

### CGZ and TGZ decrease BrdU DNA incorporation

**Figure 4:**
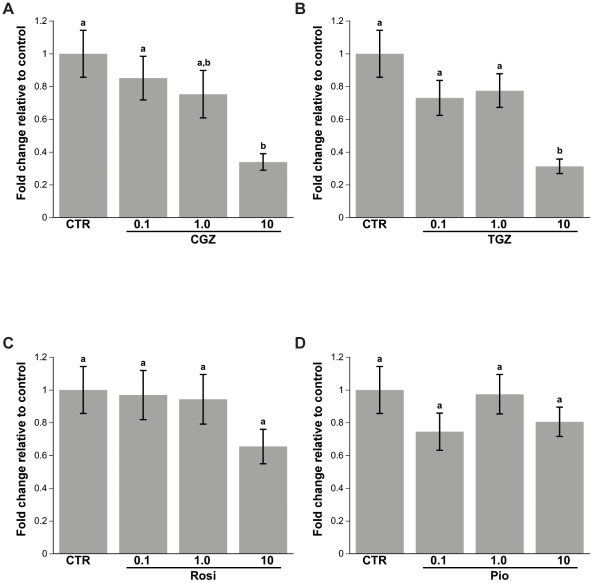
Effect of TZD treatment on Ovcar3 cell proliferation. Ovcar3 cells were serum starved for 24 hours and treated for an additional 24 hours with vehicle control (DMSO) or 0.1, 1.0 or 10 µM of CGZ (**A**), TGZ (**B**), Rosi (**C**), Pio (**D**). Cell proliferation was assessed with the BrdU assay. Results are the means ± SEM for at least 3 measurements from three individual experiments. Bars that do not share a letter designation are significantly different within a treatment group (*p*<0.05).

The MTS assay measures mitochondrial activity in cells, which is indicative of their viability [22] and considered an indirect assessment of cell proliferation. However, there is a report that the TZDs can interfere with the MTS assay [23]. Therefore, we also measured the rate of DNA replication in Ovcar3 cells using BrdU incorporation as another index of cell proliferation. Ovcar3 cells were treated with 0, 0.1, 1 or 10 µM of Rosi, CGZ, TGZ or Pio for 24 hours. BrdU assays show similar results as those of the MTS assay. There was approximately a 70% decrease in proliferation in cells treated with 10 µM of CGZ or TGZ, and no effect of Rosi or Pio on cell proliferation (Figure 4A–D).

### PPARγ mRNA expression is enhanced by TZD treatment of ovarian cancer cells

**Figure 5:**
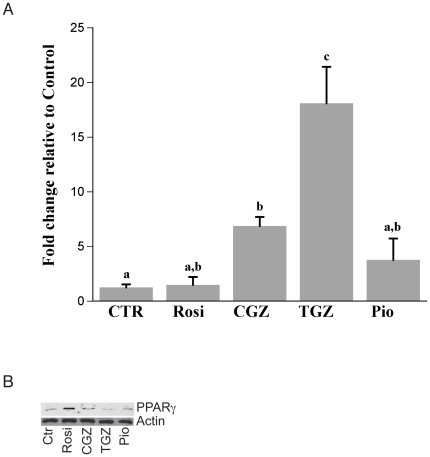
Effect of TZDs on PPARγ mRNA and protein expression. PPARγ mRNA and protein expression were measured in Ovcar3 cells using (**A**) Real time PCR and (**B**) western blot analysis after TZD treatment. Cells were serum starved for 24 hours and treated for an additional 24 hours with vehicle control (DMSO) or one of the following: 10 µM of Rosi, CGZ, TGZ, or Pio. Results are the means ± SEM for at least 3 measurements from two individual experiments. Bars with different superscripts are significantly different (*p*<0.05).

To evaluate the association of these ligands with regulation of PPARγ, we examined the effect of TZDs on expression of PPARγ in Ovcar3 cells. Cells were treated with or without 10 µM of TZDs and PPARγ mRNA and protein levels were analyzed 24 hours later. PPAR(expression was increased in Ovcar3 cells treated with CGZ (6.9 fold) and TGZ (18.1 fold) (Figure 5A). Surprisingly, there was an increase in PPAR(protein following Rosi but not CGZ or TGZ treatment (Figure 5B). In an effort to explain this discrepancy between mRNA and protein expression, we investigated a time course of PPAR(mRNA and protein expression. Cells were treated with the different TZDs for 4, 8, 12 or 24 hours and both real time PCR and Western blot analysis were performed. We did not observe a correlation between the expression patterns for PPAR(mRNA and protein across time which indicates that there are potentially other mechanisms affecting the levels of PPAR(protein in these cells. One possibility is that the TZDs increase the turnover and degradation of PPAR(protein as seen in other systems [24], [25].

Since CGZ and TGZ caused a decrease in cell proliferation in CaOv3 and Skov3 cells, we measured the mRNA expression of PPARγ in these cells. In CaOv3 cells, the mRNA expression of PPARγ after CGZ and TGZ treatment was 3.6 and 4.4 fold above the vehicle control respectively although these changes did not reach significance. Skov3 cells showed no changes in PPARγ expression after treatment with the TZDs (data not shown). Collectively, our data indicates that PPAR(mRNA is present and is regulated by TZDs in ovarian cancer cells albeit to different degrees depending upon the cell type and the ligand used to activate PPARg.

### CGZ and TGZ do not induce apoptosis

**Figure 6:**
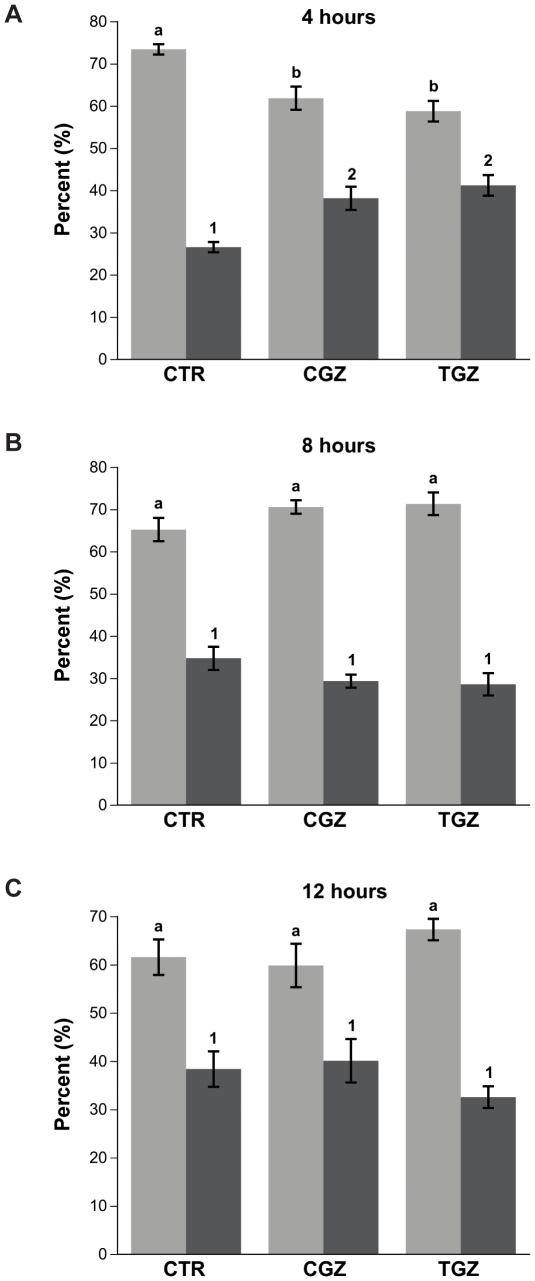
Annexin V assay to detect apoptosis in Ovcar3 cells. Ovcar3 cells were serum starved for 24 hours and treated with vehicle control (DMSO) or 10 µM of CGZ or TGZ for (**A**) 4 hours, (**B**) 8 hours, or (**C**) 12 hours. Results are the means ± SEM of 3 measurements from three individual experiments. Light gray bars represent viable cells; dark gray bars represent cells undergoing early and late apoptosis as well as those that are dead. Bars that do not share a letter or number designation are significantly different (*p*<0.05).

In this and subsequent experiments we examined only Ovcar3 cells as these cells showed the most notable effects when treated with TZDs. Furthermore, these cells are commonly used in the study of ovarian cancer, facilitating comparison with previous studies. In an attempt to better understand the mechanism mediating the decrease in cell proliferation, we examined whether apoptosis contributes to this decrease. We observed a decrease in cell proliferation after 24 hours of treatment (Figures 3 and 4). If this decrease is due to apoptosis, then this cell death should have occurred at an earlier time point. Hence, we examined the effect of TZDs on Ovcar3 cells and determined whether cells were either viable or undergoing apoptosis and were dead at 4, 8 or 12 hours after treatment using FACS analysis. There was a slight increase in apoptotic and dead cells after 4 hours of treatment with CGZ and TGZ (Figure 6A). However, this increase was not detected in samples treated for 8 or 12 hours (Figure 6B and 6C). Trypan blue experiments to confirm live versus dead cells showed similar results (data not shown).

### CGZ and TGZ cause cell cycle arrest

**Figure 7:**
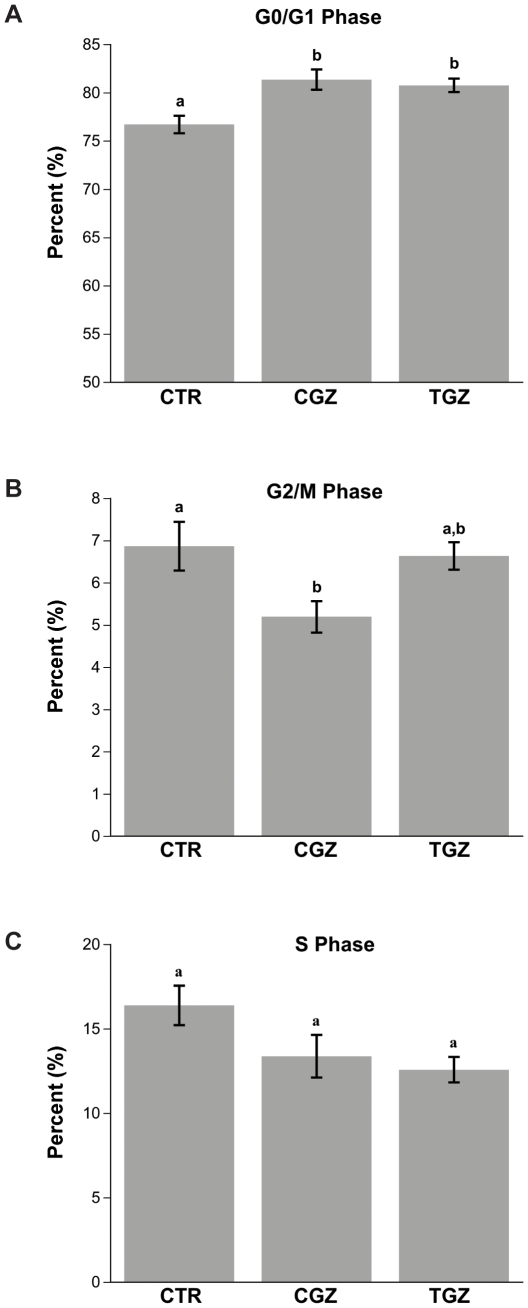
Cell cycle kinetics using flow cytometry. Ovcar3 cells were serum starved for 24 hours and treated for an additional 24 hours with vehicle control (DMSO) or 10 µM of CGZ or TGZ. (**A**) G_0_/G_1_, (**B**) G_2_, (**C**) S phase. Results are the means ± SEM for at least 3 measurements from three individual experiments. Bars that do not share a letter or number designation are significantly different (*p*<0.05).

In an effort to clarify the reason underlying the decrease in proliferation, the effects of TZDs on cell cycle progression were also assessed. A significant increase in cells in the G_0_/G_1_ phase was seen following treatment with TGZ and CGZ (Figure 7A). CGZ administration resulted in a significant decrease in G2/M phase of the cell cycle (Figure 7B), while no changes in the S phase were seen in cells treated with CGZ or TGZ (Figure 7C). Cells treated with Rosi or Pio did not show any change in cell cycle distribution compared to control (data not shown).

### Effects of TZDs on PPRE luciferase activity

**Figure 8:**
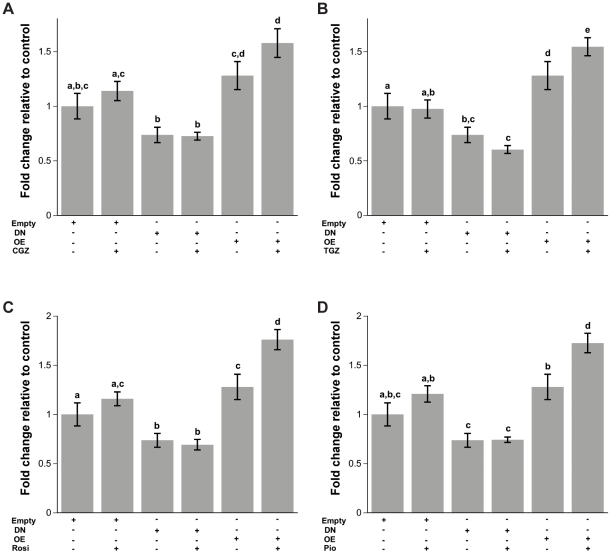
PPRE Luciferase promoter activity assay. Ovcar3 cells were double transfected with a PPRE construct and one of the following: Empty vector, DN, or OE PPARγ. After the double transfections, cells were treated for 24 hours with vehicle control (DMSO) or 10 µM of (**A**) CGZ, (**B**) TGZ, (**C**) Rosi, (**D**) or Pio. Results are the means ± SEM for at least 3 measurements from three individual experiments. Bars that do not share a letter designation are significantly different (*p*<0.05).

To clarify whether the antiproliferative and cell cycle arrest effects seen with select TZDs are a direct effect of PPARγ transactivation, we transiently transfected cells with a 3XPPRE-mTK-pGL3-reporter plasmid. Two additional constructs were cotransfected, namely, a DN form of PPARγ and an overexpression (OE) of a wildtype PPARγ construct. Cells were then treated for 24 hours with 10 µM of Rosi, CGZ, TGZ, or Pio. None of the TZD treatments alone increased PPRE activity (Figure 8). However, cells transfected with DN showed an approximate 40% decrease in PPRE mediated activity in both untreated and TZD treated cells. Furthermore, increasing the expression of the wild type PPARγ showed an increase in luciferase activity when cells were treated with any of the four different TZDs compared to cells that were only treated with TZDs (Figure 8A–8D).

### Analysis of TZD mediated PPARγ dependent and independent actions

**Figure 9:**
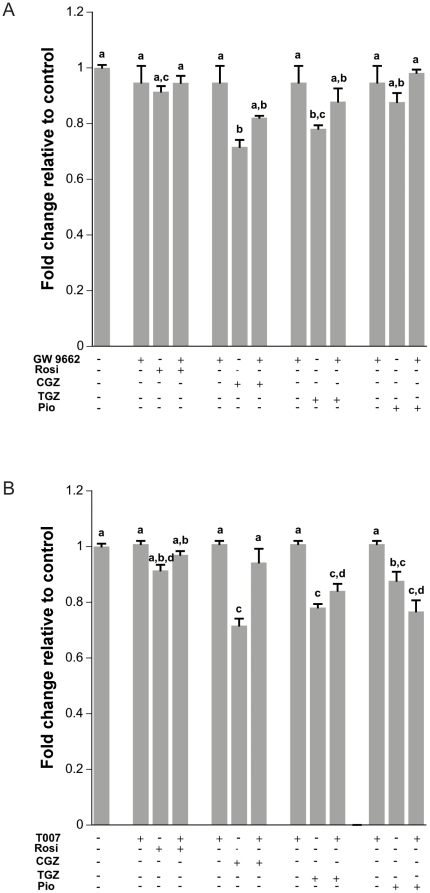
Effect of PPARγ antagonists on Ovcar3 proliferation. Cells were treated with GW9662 (**A**) or T007 (**B**) for 24 h and cell proliferation was assessed. Results are the means ± SEM for at least 4 measurements. Bars that do not share a letter designation are significantly different (*p*<0.05).

In order to determine whether the effects of TZDs are PPARγ dependent, cells were treated with 10 µM TZDs in the absence or presence of PPARγ antagonists (GW9662, T007). The MTS assay was performed in order to determine whether the presence of antagonists could reverse the effects of TZDs on ovarian cancer cell proliferation. Results showed that there was a partial ‘rescue’ when cells were treated with the GW9662 (Figure 9A) or the T007 (Figure 9B) compounds in combination with CGZ or TGZ. However, the pattern of action was inconsistent between the two antagonists and even with the same antagonist in the presence of different TZDs. For example, T007 was able to completely block the effects of CGZ on cell proliferation but had no effect on the TGZ mediated decrease in proliferation. In addition, the use of the GW9662 or the T007 compounds alone did not affect proliferation on their own as was expected. This may be due to the fact that these antagonists may be mixed agonists or not PPARγ specific [9], [26]. These findings indicate that the use of a pharmacological approach employing reported PPARγ antagonists alone does not answer the question as to whether the effect of the TZDs act through PPARγ. This led us to use a molecular approach using DN or OE PPARγ constructs to investigate whether the effects seen on cell proliferation and cell cycle were PPARγ dependent or independent.

**Figure 10:**
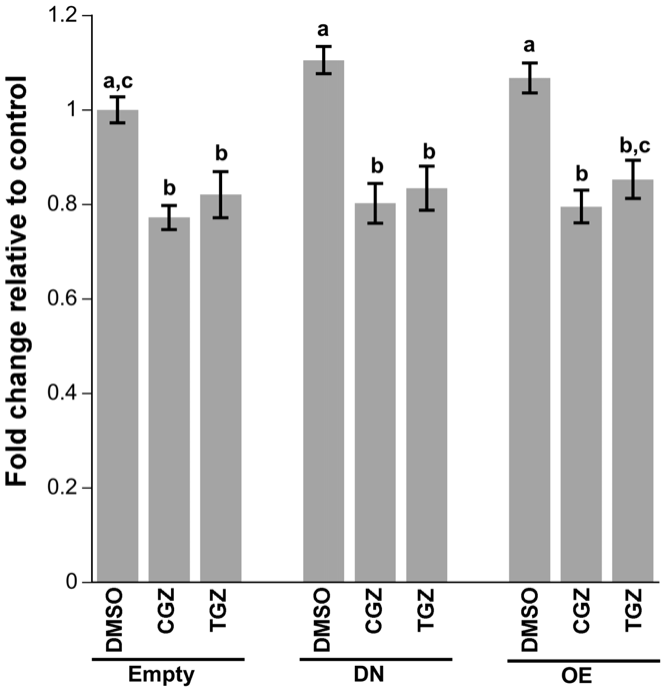
Effect of CGZ and TGZ treatment on Ovcar3 cell proliferation in cells transfected with DN or OE PPARγ constructs. Ovcar3 cells were double transfected, serum starved for 24 hours and treated for an additional 24 hours with vehicle control (DMSO) or10 µM of CGZ or TGZ. Cell proliferation was assessed with a BrdU assay. Results are the means ± SEM for at least 9 measurements from three individual experiments. Bars that do not share a letter designation are significantly different within a treatment group (*p*<0.05).

Transfecting cells with DN or OE forms of PPARγ without the presence of ligands did not change cell proliferation as indicated by BrdU assays (Figure 10). More importantly, the effects of CGZ and TGZ on proliferation were not overcome by the presence of the DN form of PPARγ (Figure 10). Luciferase assays were run on samples in the same plates in order to ensure that cells were being transfected and expression of the DN caused the expected decrease while overexpression of wild type PPARγ increased PPRE activity, respectively (data not shown). In concordance with the BrdU assays, cell cycle data also indicate that the effect of CGZ and TGZ are not PPARγ dependent (data not shown).

## Discussion

TZDs have been shown to have a wide range of effects on cancer cells. The presumed target of TZDs, PPARγ, is overexpressed in a variety of cancers including breast, lung, colon, prostate and ovary [2], [27], [28], [29]. Although, the exact role TZDs and PPARγ play in ovarian cancer remains unknown, this study demonstrates that CGZ and TGZ have anti-proliferative actions on three ovarian cancer cell lines, whilst Rosi and Pio have no effect. The fact that these anti-proliferative actions are seen in all three cell lines suggests that this is a common phenomenon and not confined to a single ovarian cancer cell line. The experiments described herein also demonstrate that the four TZDs have distinct actions on ovarian cancer cells possibly through both PPARγ dependent as well as independent pathways. Although several reports indicate that different cell lines respond differently to TZDs, [30], [31], [32], [33], to our knowledge, a direct comparison between these four TZDs in ovarian cancer and delineating whether these effects are PPARγ dependent using molecular, physiological and pharmacological approaches has not been investigated.

In the present study, 10 µM of CGZ and TGZ decreased cell proliferation in all three of the ovarian cancer cell lines studied. These observations are in concordance with other studies utilizing ovarian cancer cells as well as other cell lines where CGZ and/or TGZ decreased MTT activity [20], [21], [34]. However, our findings are in contrast to cancer cell lines from other tissues such as breast, thyroid, bladder and others that usually require much higher concentrations of TZDs to elicit a biological response. In fact, doses up to 100 µM, which exceed the reported ligand specific concentrations of 10 µM or less [12], may be required before an inhibition of proliferation is seen [35], [36], [37], [38], [39]. However, these differences may be due in part to the use of different cells lines or differences in cell culture conditions where, for example, investigators have used 1% FBS [39], 5% FBS [35], [36], [37], [38], [39] or 10% FBS [21]. The present findings demonstrate that this decrease in proliferation is due to an increase in cell cycle arrest rather than an increase in apoptosis. Similarly, other reports have shown that TZDs lead to the degradation of cyclin D1 in prostate cancer [40], which causes cell cycle arrest, and a decrease in tumor cell proliferation. Likewise, in A2780 ovarian cancer cells, CGZ decreases cyclin D1 along with other pro-survival factors resulting in cell cycle arrest [21]. Yang and colleagues [20] reported that CGZ and TGZ treatment of ES-2 and PA-1 ovarian cancer cells resulted in cell cycle arrest; however, this change in cell cycle kinetics was associated with increased levels of apoptosis. Although our studies support the previous findings in different ovarian cancer cells that the TZDs decrease cell proliferation by arresting cells in the G0/G1 stage of the cell cycle, the possibility exists that the higher concentrations of TZDs used in these previous studies results in the induction of apoptosis [20].

The present study demonstrates that each of the TZDs has distinct actions on ovarian cancer cell proliferation. CGZ and TGZ cause a dramatic decrease in ovarian cancer cell proliferation whilst Rosi and Pio had no effect. This is not unexpected as each TZD has a different affinity and binding to PPAR [7], [41], recruits different coactivators [42] and may elicit distinct cellular responses. These unique actions have led to the concept that the TZDs are selective modulators of PPARγ and therefore target distinct genes resulting in tissue selectivity [12], [42], [43], [44]. In addition, TZDs have different specificities for PPARs. For example, Rosi and Pio are considered the most potent PPARγ agonists out of the four TZDs used in this study [11]. The findings that these potent PPARγ agonists do not inhibit cell proliferation further support our hypothesis that the actions of the TZDs may be independent of PPAR. Furthermore, Rosi, CGZ and TGZ are specific to PPARγ while Pio has been shown to also activate PPARα [45], [46]. Hence, our data may reflect the differences in the selective modulation of PPARγ, differences in the specificity for PPARγ, or the activation of distinct PPARγ independent pathways.

To begin to understand the selective modulation of PPARγ by TZDs in ovarian cancer cells, we examined the mRNA and protein expression profiles of PPARγ and the activation of PPARγ's promoter following TZD treatment. There is a dramatic increase in PPARγ mRNA expression when Ovcar3 cells were treated with TGZ and to a lesser extent with CGZ and Rosi. In contrast, the highest expression of PPARγ protein was observed when cells were treated with Rosi. This discordance between the expression of PPARγ mRNA and protein after TZD treatment has not been observed nor examined previously, however, the levels of PPARγ protein after TZD treatment have been shown to be highly variable between ES-2 and PA-1 ovarian cancer cells [21]. We postulated that examination of a static 24 hour time point might not accurately capture the induction of PPARγ mRNA and protein. We therefore assessed the level of PPARγ mRNA as well as protein patterns at different times after TZD treatment and observed a lack of correlation between PPARγ mRNA and protein expression at all time points. An alternative theory to explain this discordance is that the increase in expression of PPARγ mRNA when cells are treated with TGZ may increase the turnover and degradation of PPARγ protein thereby reducing protein expression. This has been previously demonstrated in other systems where it was shown that with increasing doses of TZDs, there was an increase in PPARγ protein degradation which resulted in a decrease in the steady state levels of the protein in endothelial cells [24], [25]. This is hypothesized to occur because TZDs mediate changes in the phosphorylation state of PPARγ by MEK which leaves PPARγ susceptible to degradation [47]. In light of this information, our protein data could be explained by the fact that TZDs cause differential degradation of PPAR(after activation and that the turn-over rate of PPAR(protein in cells treated with TGZ is faster than that of CGZ and Rosi resulting in a decrease in protein levels. In addition, it is possible that Rosi increases the protein stability of PPAR(whilst the effects on proliferation after treatment with CGZ and TGZ are PPAR(independent. Alternatively, TZDs may cause changes in the protein itself, such as phosphorylation, which alters other actions of the protein rather than just changing its overall transactivation as recently been shown by Choi et al. [48].

In light of our results that showed a lack of correlation between the levels of PPAR(mRNA and protein after TZD treatment, we explored the changes in activation of a PPAR(reporter as an index of PPAR(activity after TZD exposure. Surprisingly, PPAR(activity was not stimulated by any of the 4 TZDs after 24 h of treatment, in contrast to previous reports in other ovarian cancer cell lines that 50 mM of CGZ increased luciferase activity of a PPRE reporter by 18 h [21]. Based on this previous report, we speculated that promoter activation might be occurring at earlier or later time points. Hence, we tested the effect of TZDs on promoter activity at 4, 8, 24 and 32 hours. There were modest increases in luciferase activity in cells treated with CGZ at 8 and 32 hours (data not shown), which were insufficient to account for the changes in PPAR(expression. Discordance between protein levels and PPAR(activity has been previously seen in breast cancer cells but the exact mechanism underlying this difference remains unknown [34]. However, such an observation suggests that analysis of only PPARγ protein levels may mistakenly be used to attribute cellular effects or responsiveness to treatments.

The TZDs did not increase endogenous PPAR activity in these cells, however, TZD treatment increased luciferase activity when PPARγ was overexpressed in Ovcar3 cells. This indicates that these cells constitutively activate PPARγ and, when the receptor is highly abundant, further activation is possible. This activation is not limited by RXR, since 9-*cis*-RA does not affect the PPRE activity in Ovcar3 cells when transfected with empty, DN, or wildtype PPARγ vectors. The difference in endogenous ligands or activating capacity between cells could lead to an increase in endogenous transactivation and potentially, an increased receptor degradation rate. Therefore, cellular levels of PPARγ protein do not reflect the total activity of PPARγ or the capacity of exogenous ligands to overcome endogenous PPARγ activation.

The present study explored whether proliferation and cell cycle arrest observed after TZD treatment were due directly to PPARγ. Previously, the PPARγ antagonists T007 and GW9662 were used to determine whether the effects of TZDs are PPARγ dependent [20], [49]. Our results showed no changed in cell proliferation when cells were treated with these two antagonists alone. When cells were treated with the antagonists in combination with TZDs, there were differential changes in cell proliferation. However, there are controversies regarding the specificity of PPARγ antagonists [9], [26], in particular that they can activate, rather than inactivate the PPRE reporter in normal breast cell lines [9]. Antagonists may also have PPARγ independent effects [26]. Due to these potential confounding non-PPARγ actions of the PPARγ antagonists, we used an alternative molecular approach to determine whether TZDs act through PPARγ. To accomplish this, we transfected Ovcar3 cells with a dominant negative form of PPARγ or a wild type overexpression PPARγ construct. The DN form of PPARγ provided a more robust decrease in PPRE when compared to shRNA (data not shown). The observation that the DN decreased PPRE promoter activity again illustrates that there is endogenous transactivation of PPARγ taking place in these cells. In addition, the finding that the TZDs cause an increase in reporter activity when the level of PPARγ is overexpressed demonstrates that TZDs convey part of their activity through PPARγ when there are sufficient, unoccupied receptors available. One can argue that the effect on proliferation may be due to the TZDs competing for the receptor with the endogenous ligand and recruiting different co-activators [42]; however, all four TZDs showed a similar trend in terms of luciferase activity indicating the possibility that they may convey some actions through PPARγ but the effects on proliferation may not be PPARγ dependent. This was evident when Ovcar3 cells were concurrently transfected with the wild type or DN PPARγ, treated with CGZ or TGZ, and cell proliferation still decreased, indicating that the effects of CGZ and TGZ are not PPARγ dependent. However the possibility exists that, even in the presence of the DN, there is still sufficient PPARγ to impact cell proliferation. Irrespective, this approach provided evidence that there was a lack of an effect of endogenous PPARγ on cell proliferation since the expression of a DN or OE form of PPARγ did not affect cell proliferation.

In summary, our findings demonstrate that CGZ and TGZ decrease cell proliferation mainly through cell cycle arrest. The current study establishes that these effects appear to be TZD specific as these changes are not seen in cells treated with Rosi or Pio, unlike previous reports [20], [21], yet all four TZDs drive the same levels of PPRE activity. This could be due to several hypothetical explanations. Rosi and Pio may be recruiting different coactivators than those of CGZ and TGZ. Alternatively, the effects of TZDs on proliferation may be via changes in the Akt/PTEN/mTOR pathway [50]. Rosi has been previously shown to suppress the AKT pathway via increasing the expression of PTEN in cells such as MCF7 [51] and NSCLC [27]. Thus, it is possible that CGZ and TGZ cause a more pronounced effect on the AKT/mTOR pathway than Rosi and Pio. Another plausible explanation is that variations in the specificity of these ligands for PPARγ, thus, causing changes in promoter activity such as a prolonged DNA binding activity rather than an increase in magnitude. Alternatively, the changes in cell proliferation with CGZ and TGZ may be independent of PPARγ. For example, adding DN in combination to CGZ or TGZ did not rescue Ovcar3 cells from the decrease in proliferation, indicating that these compounds are at least partially acting in a PPARγ independent manner.

Our findings also demonstrate that PPARγ agonists stimulate PPARγ expression, but to varying degrees in different ovarian cancer cells. This stimulation of PPARγ mRNA is not mirrored by PPARγ protein levels and interestingly, the levels of PPARγ protein do not accurately predict PPRE reporter activity. These data suggest that TZD actions may be beneficial in hindering the proliferative capacity of tumor cells albeit potentially in a PPARγ independent manner. This observation is the first report to our knowledge that utilized both molecular and pharmacological approaches to demonstrate that TZDs convey their actions on ovarian cancer cells independent of PPARγ. Since TZDs have often been suggested as plausible therapeutic agents due to their effects on proliferation and antitumor potential in ovarian cancer [52], it will be crucial to further investigate potential targets of TZDs in order to understand their mechanism of action and uncover novel therapeutic targets.

## Materials and Methods

### Ethics Statement

This study does not involve human participants or animal work. As such, an ethics statement is not required.

### Cells, Media and Reagents

All cell lines (Ovcar3, CaOv3, Skov3 and MCF7) were obtained from the American Type Culture Collection ATCC (Rockville, MD). Ovcar3, CaOv3 and Skov3 cell culture media were obtained from the American Type Culture Collection ATCC (Rockville, MD). Ovcar3 cells were grown in Rossman-Park-Memorial-Institute (RPMI) 1640 media supplemented with 20% fetal bovine serum (FBS) containing penicillin/streptomycin/amphotericin B (Gibco-Invitrogen, Carlsbad, CA). CaOV3 and Skov3 cells were grown in Dulbecco's Modified Medium (DMEM) and McCoy's 5A medium respectively. MCF7 media (DMEM-31053) was purchased from Invitrogen. DMEM, McCoy's and MCF7 media were supplemented with 10% FBS and antibiotics as above. Ciglitazone (CGZ), Rosiglitazone (Rosi), Troglitazone (TGZ), Pioglitazone (Pio) GW9662 and T007 (T0070907 (N-(4′-aminopyridyl-2-chloro-5-nitrobenzamide)) were purchased from Cayman Chemicals (Ann Arbor, MI), while 9-*cis-*retinoic acid (9-*Cis*-RA) was a purchased from MP Biomedicals (Solon, OH).

### Cell Line propagation

Cells were grown at 37°C in a 5% CO_2_ environment until cells reached the desired confluence described below. Cells were then serum starved for 24 hours and treated for an additional 24 hours with vehicle control (DMSO), or 0.1–10 µM of one of the following: Rosi, CGZ, TGZ, or Pio. The maximum concentration of TZDs used was 10 µM as PPARγ specific actions are reported with TZDs at concentrations ≤10 µM [20]. All experiments were performed as outlined above unless otherwise noted. All experiments were done at least 3 times, with 3 replicates each.

### RNA Isolation

In order to examine cellular PPARγ expression, cells were grown to 60–90% confluence and treated as described above for 24 hours. Total RNA was isolated using the RNeasy RNA isolation kit from Qiagen (Valencia, CA) according to the manufacturer's protocol. RNA samples were stored at −80°C until used for real time PCR.

### Real time PCR

The reverse transcription reaction was accomplished using the TaqMan® one step rtPCR Master Mix kit from Applied Biosystems (Foster City, CA). Quantitative PCR was performed using a Stratagene Mx3000P real-time thermal cycler and the TaqMan methodology. Pre-optimized primers for PPARγ and 18S mRNA probes with a 5′ fluorescent reporter (FAM) were purchased from Applied Biosystems. Each RT reaction was performed using 200 ng of RNA at 48°C for 30 minutes. The RT reaction product was then subjected to real time PCR by incubating the reaction at 95°C for 10 min followed by 40 cycles of denaturing (95°C for 15 s) and re-annealing (60°C for 1 min). The amount of mRNA present was recorded as a cycle number (Ct) where the message reaches a fixed threshold. Ct was normalized to ribosomal RNA 18S which was then compared to control by the equation 2^−ΔΔCt^.

### Western blot analysis

Western blot analysis was performed in order to examine the expression of PPARγ protein in the different cell lines as well as to evaluate the effect of experimental treatment on protein expression. To accomplish this, cells were grown to 60–90% confluence, treated as previously described and lysed using a lysis buffer containing a protease inhibitor cocktail (Roche, Mannheim, Germany). Protein concentrations were quantified using the Bradford assay (BioRad, Hercules, CA). A total of 50 µg of protein was run on a 10% SDS gel, which was then transferred to a nitrocellulose membrane (BioRad). Membranes were incubated with antibodies against PPARγ NR1C3 (1∶250, R&D systems, Minneapolis, MN) (Figure 1B) or PPARγ E-8 (1∶150, Santa Cruz Biotechnology Santa Cruz, CA) (Figure 6B), overnight at 4°C in 5% milk (TBST) or actin α (1∶1000, Sigma-Aldrich, A 5060) for 1 hour. Membranes were washed and incubated with secondary anti-mouse or anti rabbit horseradish peroxidase (HRP)-conjugated antibody respectively (Santa Cruz). PPARγ and actin α were visualized using an enhanced chemiluminescence detection system (Pierce, Rockford, IL) and exposure to x-ray film.

### Proliferation assays: MTS and BrdU assays

The effect of TZDs on cell proliferation was assessed with the MTS (3-(4,5-dimethylthiazol-2-yl)-5-(3-carboxymethoxyphenyl)-2-(4-sulfophenyl)-2H-tetrazolium) assay (Promega, Madison, WI), according to the manufacturer's protocol.). Briefly, after TZD treatment, cells were treated with 100 µL of MTS/PMS solution from Promega's CellTiter 96 Aqueous one solution cell proliferation assay for 4 hours. Proliferation was measured by colorimetric absorbance at 492 nm. The incorporation of BrdU into DNA was assessed using a Cell proliferation, ELISA BrdU kit (Roche). In brief, cells were labeled after culture with BrdU labeling solution overnight. Afterwards, cells were fixed, the DNA denatured, and peroxidase the BrdU complex was then detected and quantitated colorimetrically at 450 nm. Treatments were normalized to control (DMSO vehicle treatment) and expressed as the relative fold change compared to the control. BrdU proliferation was also preformed on cells that were transfected with or without a dominant negative (DN) or overexpressing (OE) PPARγ vector. Cells were double transfected as described under the constructs and transfection section below, then serum starved for 24 hours and treated for 24 hours prior to running a BrdU assay in combination with either the agonists or antagonists.

### Cell Cycle assay

Flow cytometry was used to determine the effect of TZD treatment on cell cycle kinetics, as previously described by Vindelov [53]. Cells were cultured until they were 60–90% confluent and treated as described above. Briefly, cells were washed with PBS, trypsinized for 5–10 minutes and then treated with trypsin inhibitor and ribonuclease A for 30 minutes. Subsequently, propidium iodide (final concentration 50 ug/mL) in combination with sperimine HCl was added to the cells for 30 minutes in the dark at 4°C. The suspension was analyzed using a FacsCalibur flow cytometer from Becton Dickson (San Jose, CA) at the core facility at the University of Kentucky using Mod FitLT V.3.1 software. Ratios of cells in the G0/G1, S, and G2/M phases of cell cycle were determined on the basis of their DNA content and presented as cell percentage at the end stage of the cell cycle. Cell cycle histograms were obtained from 3 determinations, each with a total of 100,000 cells/treatment. Cell cycle was also performed on cells that were double transfected with either DN or OE PPARγ. Cells were double transfected as outlined under the constructs and transfection section below, then serum starved for 24 hours and treated for 24 hours. Consequently, a cell cycle assay was performed.

### Apoptosis assay

Cells were grown as described above and treated for 4, 8 or 12 hours with one of the following: Vehicle control (0.1% DMSO), or 10 µM of CGZ or TGZ. Cells were then analyzed for apoptosis using the Vybrant Apoptosis Assay kit per the manufacturer's protocol (Invitrogen). Briefly, cells were washed twice with PBS, trypsinized and centrifuged. Annexin V Binding buffer was used to resuspend the pellet and Alexa Flour 488 annexin V and propidium iodide solution was added to the cells and incubated at room temperature for 15 minutes. Samples were analyzed using flow cytometry readings at 530 nm and >575 nm in the FacsCalibur flow cytometer at the core facility at the University of Kentucky.

### Constructs and transfection assays

PPARγ regulates gene expression by binding to a specific Peroxisome Proliferator Response Element (PPRE) [54]. To determine whether TZD treatments result in the activation of the PPARγ promoter, PPRE reporter constructs were utilized. This construct, 3XPPRE-TK-pGL3, contains three copies of the PPRE sequence (AGGACAAAGGTCA). The DN construct was a kind gift from Drs. O'Rahilly and Chatterjee (Cambridge University, U.K.) and has been modified to introduce a stop codon at amino acid 462 and has been previously characterized and used by our lab [19]. The wild type overexpression (OE) PPARγ construct was made by our laboratory. The luciferase construct lacking the PPRE and the *Renilla* constructs were purchased from Promega.

Approximately 120,000 cells (about 30% confluence) were plated per well for all transfection experiments. To compare the PPRE activity across the three different cell lines, cells were either transfected with a 3XPPRE mTK-Luc or with two vectors (a luciferase lacking PPRE and a *Renilla* containing vector) [55]. Cells were transfected two subsequent days and then lysed on day 3 for analysis. To inspect the effect of TZD treatment on PPRE activity, cells were transiently double transfected with a total of 0.4 µg DNA per well. Each well was transfected with 0.2 µg of pGL3 plasmid containing 3XPPRE mTK-Luc and *Renilla* [55] using FuGENE transfection reagent (Roche). Cells were also transfected with 0.2 µg of one of the following: Empty vector control construct, DN, or wild type overexpression (OE) PPARγ. Cell transfection was repeated for an additional 24 hours. Afterwards, cells were treated with 10 µM of one of the following: Vehicle control (DMSO), Rosi, CGZ, TGZ, Pio or 9-*cis*-RA for an additional 24 hours. Cells were lysed with 150 µl of passive lysis buffer and treated according to the manufacturer's instructions (Promega dual luciferase assay kit, Promega). A total of 20 µl was used for luminometry. Luminometry was performed on a Berthold Technologies Lumat 9507 (Willbad, Germany). Ratios between luciferase and *Renilla* were used to calculate promoter activity and adjust for differences in transfection efficiency. Transfection efficiencies were calculated and revealed that the highest transfection efficiency was achieved in Skov3 cells followed by Ovcar3 with the CaOv3 cells having the lowest transfection efficiency. Data is presented as a relative expression normalized to the vehicle control.

### Statistical analysis

All data are presented as means ± SEM. One-way analysis of variance (ANOVA) or Welch's t-test was used to test differences among treatments. If the ANOVA showed significant effects, post hoc tests were performed using Tukey's or Bonferroni, in order to identify significant differences among treatments. The means were compared with *p*≤0.05 considered significant. Statistical analysis was performed using a statistical analysis software [56].
